# Observations on the Use of a Solution of Sal Ammoniac in Vinegar, as an Application in Various Chirurgical Cases

**Published:** 1805-06-01

**Authors:** G. Wilkinson

**Affiliations:** Licentiate of the Royal College of Surgeons, &c. &c.


### *Observations on the Use of a Solution of Sal Ammoniac in Vinegar, as an Application in various Chirurgical Cases. By* G. Wilkinson, *Licentiate of the Royal College of Surgeons, &c. &c.*

AFTER what has been said long ago by the celebrated Mr. Pott,* Mr. Bromfield,† Justamond,‡ Bell,§ and others, but particularly Mr. Henry Yates Carter,|| respecting this remedy; it may, perhaps, be deemed not only a superfluous, but an unnecessary task, to say more on this subject; more particularly, as it already appears to be so well known and fully understood.

Admitting, however, the full force of these objections generally, it does not appear, that the inquiry we are about to make respecting this useful application is exhausted; and the more so, as it seems not to have been noticed in any separate treatise with the distinction to which it seems entitled, if we except what has been related of its wonderful effects in lacerated wounds, by Mr. Carter, which, from my own experience I can say, he has not exaggerated.

If it has not been deemed unimportant, or uninteresting to enter into disquisitions on the effects of cold water,¶ warm and cold bathing,** riding on horseback,†† sea voyages,‡‡ use of the dumb bells, swinging,§§ friction,|||| electricity,¶¶ and various other apparently simple operations that have been found eminently beneficial to mankind; this attempt of mine to extend the use of this remedy more generally than heretofore, may, perhaps, prove not altogether useless.

Thirty years ago, or thereabouts, (and perhaps too much so, even now) it was generally the practice, in almost

---

* See his Chirurgical Works.

† Observations and Cases in Surgery, vol. 2.

‡ On Cancerous and Schirrhus Disorders.

§ System of Surgery and Treatise on Ulcers.

|| Med. Facts and Observations, vol. 2, p. 14; vol. 6, p. 66.

¶ Vide Dr. Currie's Reports.

** Sir J. Floyer Baynard, Dr. Russel, &c.

†† Dr. Sydenham's Works.

‡‡ Dr. Gilchrist.

§§ Dr. Carmichael Smyth

|||| Hippocrates, Boerhaave's Medical Correspondence, &c.

¶¶ Priestly, Cavallo, Birch, Lowndes, &c. &c.

every accident, whether of simple or compound fractures, dislocations, sprains, bruises, lacerated wounds, and even in common cuts, or incised wounds, to apply warm emollient poultices: nay, scarcely was any operation in private practice, or public hospitals performed, were they ever so important or trifling, but these were (and still are, by many) the first applications used; and it also was the practice in many other cases of surgery, as erysipelas, inflammations of the eyes, kibes, chilblains, tetters, ringworms, and even in some other cuticular eruptions.

Mons. Goulard, about this period, having written his valuable dissertation on lead,* and, besides demonstrating its surprising effects as a sedative, clearly pointed out the bad consequences of emollient applications in general; and moreover, having strongly urged the absolute necessity of applying his remedy, whether in lotion or cataplasm, (except in some particular cases, as contractions of the joints, indolent tumours, &c.) for the most part cold, excited the inquiry of medical practitioners so far as to adopt his plan, which, by experience, they found far preferable to that which had been heretofore practised.

Hence the diluted *aq. litharg. acetat.* became a favourite remedy, and was very generally adopted.†

In many instances, however, when used indiscriminately, it proved of no essential service, while in *very few* others the absorption of the metallic particles of the lead proved hurtful; yet, by its superseding in a great measure the pernicious use of warm poultices, stimulating and emollient fomentations, &c. the treatment of many parts of the chirurgical art was much improved, and more safe and less tedious cures, in very many instances, were effected.

It is, I think, almost needless to observe, that in most accidents where much violence ensues, as fractures, dislocations, sprains, bruises, lacerated wounds, &c. inflammation and vehement pain, if they do not immediately take place, follow sooner or later, according to the degree of injury inflicted; and when this is the case, symptomatic fever, more or less, is the consequence.

To prevent this evil is as much the province of surgery, and

* See his Treatise on Lead, 1770.

† This was greatly owing to the recommendation of Mr. Benj. Bell, whose experience and judicious remarks on its use, strongly pointed out its merits. See his Treatise on Ulcers, &c.

and perhaps more than medicine: the disease inflicted here is local; hence it follows, that local applications are obviously the primary object; and as we are now become better acquainted with the laws of the animal economy, a more rational treatment has in general been adopted, the use of warm relaxing poultices and fomentations, at least in the first instances of these injuries much lessened; except indeed in some cases, where it still prevails to my certain knowledge, not only in private practice, but even in some public hospitals.

If this injudicious custom continues through the effect of ignorance, it is much to be lamented; but if persisted in through the force of habit, it is with the sole view of pointing out the disadvantages attendant on this treatment, that I have been induced to offer these remarks.

Taking it for granted in a general way, that the use of emollient cataplasms and warm fomentations to recent injuries are pretty generally laid aside, and that the use of the diluted *aq. litharg. acetat.* is found more beneficial, particularly in cases where the parts are not wounded, which in these instances are usually of a less dangerous nature; yet, as there is some risque of mischief from the absorption of the metallic particles of the lead where there is a wound, many practitioners have been on this account induced to lay aside saturnine applications: now, while some prefer vinegar alone, or mixed with water, others have thought it better to add to it the crude sal. ammon. and that this is certainly preferable to the *aq. saturn.* or vinegar alone, or mixed with water, myself, as well as many others, will admit.

This medicine is penetrating, discutient, and less stimulating than the *spt. vin. camph. linim. sapon, &c.* it diminishes inflammation more rapidly, eases pain, and is by far a less expensive remedy: hence it is to be preferred, not only as being more certain, but because it is more simple, and perhaps less liable to do injury. Crude *sal. ammon.* dissolved in vinegar, provided it is not too much saturated with the former, appears to act as a sedative, and is devoid of any strong stimulus, except when applied to parts in a very irritable or inflamed state, or, as I before observed, too strongly saturated with the salt.

I pretend not to enter into any disquisition or inquiry into the chemical properties of this application, or to account for its action on the parts to which it is applied in an experimental way by torturing brute animals; which,

 however however useful they may appear to ascertain some doubtful points in physiology, could by no means have produced any thing satisfactory for this purpose: but, as I found a Mons. Paul, mentioned by Goulard in his treatise,* has by experiments made on the coagulating lymph of the blood, found that *solutio sal. ammon.* more readily dissolved it than the *ext. saturn, sal. nitri*, or *aq. calcis*, I repeated them on the coagulum of blood and albumen ovi, and found that this solution acted more powerfully, by rendering both of them more soft and tender than the abovementioned substances; the sal nitri was used in a state of solution in vinegar of the same strength as the sal ammon. and neither the extr. saturni nor aq. calcis was diluted. Now, although I do not say that from experiments instituted on dead matter, we are to draw conclusions analogous to what may take place in the living body; yet it would appear from hence, that the sal ammon. solution has no tendency to coagulate, but rather to dissolve gelatinous fluids, not from these experiments alone, but particularly from its general effects as an application to parts containing extravasated fluids; such as ecchymosis, bruises, sprains, fractures both simple and compound, and in some cases of white swellings in the joints proceeding from violence that are not already in a state of suppuration, or where contractions of the tendons and muscles have not taken place, forming a sort of stiffness, or in a diseased state of the articular cartilages; in these last some other mode of treatment must be adopted, which it is not my business here to relate.

The wonderful and sudden effects of cold water pumped or poured at a distance on *recent* sprains, bruises, and even fractures, by its diminishing tumefaction, easing pain, and suppressing inflammation, must be known and witnessed by some practitioners; and few who have been in the habit of applying it in such cases, cloths dipped in the diluted *aq. litharg. acet.* quite cold, and frequently repeated, must have, though in a lesser degree, experienced similar effects. But then it must be recollected, that although the inflammation and pain will in may instances go off, yet a hardness or stiffness, and sometimes numbness remains, the removal of which requires a different treatment: a warm poultice may, and is sometimes used; but

---

* See Goulard's Treatise, p. 182 and 197.

but most assuredly for reasons already and hereafter to be mentioned, is none of the best applications.

The acetated *solutio sal ammon. crud.* will, however, generally answer these purposes (if not better than the affusion of cold water, which last must be applied instantaneously after the reception of the injury) at least preferable to the diluted *aq. litharg. acet.* and if applied at first the better; though even long afterwards, when inflammation is formed, it appears superior to any, or perhaps all other modes of treatment; and if repeatedly and constantly persevered in for a time, the pain, tumefaction, and inflammation not only go off quickly, but the hardness, stiffness, or numbness will be prevented, the extravasated fluid completely absorbed, and, if no destruction or derangement of the organization of parts has ensued from the accident, suppuration will be prevented, and a complete cure effected by the first intention; the truth of all this I have repeatedly and constantly experienced for some years; so that to narrate a series of cases would only be relating what I think requires no great faith to believe, as being already demonstrated.

But, as a striking instance lately came under my observation, in which the stages of the effects of the different applications I have referred to were particularly exemplified, I shall relate it, in the order in which it began and terminated.

A healthy man, thirty years of age, received a blow in the direction of the ulna of the left arm; pain, tumefaction, and some degree of inflammation took place. A medical practitioner advised a warm poultice, which he used for four or five days; the pain being increased and tumefaction enlarged, he was afterwards recommended to consult me, by a man who had suffered most grievously from the effects of warm poultices, recommended to him by the same gentleman. I found an extravasation which occupied two-thirds of the length of the bone, with which it appeared to be in contact, and this extended to the olecranon. I immediately reversed the treatment, by causing him to apply constantly and repeatedly, the diluted *aq. litharg. acetat.* in the form of a poultice made with white bread, and applied quite cold; this was used for several days: the pain very soon abated, the inflammation seemed quite gone, but a stiffness of the parts remained, together with a visible and palpable collection of fluid in contact with the olecranon, near its articulation with the os brachii. Anxious to prevent suppuration, I thought

 of of the acetated *solutio sal ammon.* which was used, and the next day he was visibly better; the swelling being diminished, he continued it for a week or ten days, at the end of which time the extravasation was completely absorbed.

The great number of applications usually recommended on these occasions, such as linim. sapon, alias opodeldoc; spt. vin. camph. lin. volatile, Riga balsam,* bals. sulph. terebinth. or what is the same, Haerlem oil, otherwise named cripple oil; British oil,† ol. terebinth. essence of mustard,‡ and various other equally stimulating or relaxing remedies, among which may be included warm poultices and fomentations; are so contradictory in their nature, and widely different in their operation and effects, that it appears very doubtful whether this subject is clearly understood. A careful attention, however, to their obvious action or effects on the parts, or accidents to which they are applied, so far as I have been able to ascertain this by observation, is what I shall attempt to relate; and by leaving it to the reflection of others, whose experience may enable them to judge rightly, we may, I think, be led to discriminate more accurately concerning their respective merits.

The principal design of the surgeon being, in all recent accidents, to moderate pain, retard inflammation, and, if possible, promote absorption; whereby union, by the first intention, is speedily effected, cannot well be accomplished by any of those means which strongly tend to stimulate the parts to increased action; and that this is not liable to be produced by the applications just now mentioned, will appear obvious to those who have carefully attended to their effects.

That

---

* This remedy, which is imported from Riga, appears to be common rectified spirits; it is of a light greenish colour, and slightly bitter taste: its great reputation and demand among the common people, has caused it to be imitated in this country, which is effected by tinging rectified spirits with the juice of walnut leaves.

† Balsam of sulphur is composed of the latter ingredient, dissolved by heat in the essential oil of turpentine. The Haerlem oil, which was formerly imported from Holland, and that under the name of British oil, is, in fact, the very same.

‡ A name very artfully applied to what evidently appears to be a composition of essential oil of turpentine, in which camphor is dissolved, and slightly tinged with lintseed oil. Essence, or oil of mustard, is totally void of the pungency or stimulus of the seed. It seems, however, an useful remedy in old sprains, chronic rheumatic pains, and slight paralytic affections, &c.

That this is also the practice of many not versed in the laws of the animal economy, and ignorant of the *modus operandi,* as well as properties of the applications they are using, is a fact that cannot be controverted; as by these means they mechanically, as it were, apply in the first stage of the accident, that which should have been used in the middle, or latter end.

The general manner in which such remedies as the aqua saturn. acetated solution of crude sal ammon. and spt. Mindereri, seems to have been, in surgical cases, recommended by Messrs. Pott, Bromfield, &c. has led me, and no doubt others, to suppose any of them might prove equally successful, and that not only in contusions, sprains, fractures, &c. but other cases that might require the same kind of treatment. This, which I believe may be found true in general practice, has notwithstanding induced me to wish to distinguish the solution of the crude sal ammon. from other applications, for which I shall beg leave not only to point out my reasons, but endeavour, as far as I am able, to ascertain from facts and experience, the different cases and situations in which these applications, as well as others that are recommended, ought, or ought not, to be applied; noticing, at the same time, the degree of strength of the solution, according to the nature of the cases.

Now, as some are averse to saturnine applications, where there is danger from absorption, their use may be omitted in all solutions of continuity; as in fractures, dislocations, contusions, &c. accompanied with laceration, and indeed in wounds or ulcers of any sort. But in all those proceeding from accidents, whether incised or lacerated, and also in accidents where no solution of continuity takes place, the sal ammon. solution applied particularly on the first onset, will prove beyond doubt a most admirable remedy. No one, however, whom I know, has ventured to apply it so generally, and in the form of a dressing, if we except the ingenious Mr. Henry Yates Carter,* surgeon, at Kettly in Shropshire. The proofs he has adduced in its favour by the recital of several desperate and even hopeless cases of contusion, bruises and fractures, accompanied with extensive lacerations, &c. induced me to give it many fair trials in similar cases; and in every instance in which it was applied, the pain, where the wound was considerable,

 proved

* Vide Medical Facts and Observations, vol. 2 and 6.

proved somewhat violent, yet this never continued for more than a few minutes.

So very opposite are the effects of this treatment in the cases we have named to that of warm poultices, fomentations, and even the diluted *aq. litharg. acetat.* that instead of tumefaction and pain arising, which most generally occur in a greater or lesser degree from the consequent inflammation, I found the swelling which had taken place in the time prior to its use, entirely subsided by the next day; and in cases where the integuments had not been much bruised, and their structure or organization not destroyed, they very shortly after were frequently united by the first intention; while in those that had been greatly injured, scarcely less time than three, or at most four days elapsed till a mild suppuration would come on; which, to my great surprise, was usually less time than I had hitherto observed to take place on former occasions under a different treatment.

In a case which occurred to me in a seamen, named Alexander Ross, 33 years of age, the effects of this application were astonishing: his arm, just below the insertion of the deltoid, and across the belly of the *biceps, brachæus internus,* and *triceps* muscles, was squeezed between the ship's side and anchor stock; the bruise was such, that it appeared nipped close to the bone; a considerable tumefaction, with ecchymosis, immediately took place, and some discharge of blood from a small wound on the interior part of the arm, in the *biceps* muscle, afterwards followed. Fearful that the *arteria brachialis circumflexa,* or some other collateral branches, might have been so much injured as to render necessary an operation, I was somewhat alarmed, though I immediately provided for that purpose. But, by plentiful venæsection from the arm on the opposite side, and the constant and repeated application of compresses dipped in the acetated *solutio sal ammon.* I had the superlative pleasure of perceiving the tumour subside gradually, and that rapidly for the first 24 hours. A constant discharge of fluid, tinged with blood, continued to proceed from the wound for the first two or three days; this afterwards became of a lighter colour, at length yellowish and transparent. It continued to ooze in this manner for about fifteen days, when the arm appeared to be nearly reduced to its natural size, but was much smaller on the part nipped. The cure was effected in about four or five weeks, except a very small discharge of pus from the wound, which was nearly closed.

It

It was no small pleasure to perceive, that no suppuration or any tendency to this termination took place; and this circumstance surprised me the more, as I expected the great injury which the muscles and exterior covering seemed to suffer, would most probably terminate in this process.

I do not say that suppuration would have occurred had warm emollient cataplasms been used; but I must aver, that this treatment would not only have promoted it, but at any rate rendered the cure more tedious.

If I may be permitted to hazard my conjectures on this case, I would say, that the solvent, or, if you please, penetrating and discutient property of this application, assisted the absorbents so effectually, as enabled them very speedily to convey back into the vascular system the coagulable lymph and red particles of the blood extravasated; for very little pure blood was discharged, except on the first reception of the accident. No symptomatic fever took place in any considerable degree; his pulse was somewhat more quick than usual, and such a degree of pain ensued for four or five days, as required an anodyne at bed-time. Throughout the whole process of his cure, no other application was used but the solution, except at last a little sticking plaster to cover the small wound, after it had ceased discharging ought else than a very trifling quantity of purulent matter.

A most distressing accident took place in the month of August, 1800; John Goodwin, a sailor lad, sixteen years of age, had the middle and inner part of his right thigh bruised between the ship's side and sharp edge of the anchor. The external integuments, *vastus internus,* and part of the *Sartorius* muscles were partially divided; the wound was more than four inches in length across, and about one inch and half wide; a considerable hæmorrhage ensued from this wound; immediately below the articulation of the joints of the inner ankle, where it unites with the astragalus and bones of the tarsus, the integuments and muscles were torn downwards, the tendo Achillis was visible, although the bones were not bare, nor their articulating ligaments, or blood vessels supplying the foot divided.

Some sutures were applied to both wounds, so as to retain their integuments as near in contact as could be without being tight; slips of adhesive plaster were also applied with lint, and compresses moistened with sal ammon. solution, and these retained by light bandages; the whole of of this covering was kept constantly moist with the solution, and anodynes were given occasionally. At the end of three or four days, when inflammation had come on, which in this case was not *inconsiderable,* though unattended with much *tumefaction,* I clipped off the stitches; the integuments had assumed a gangrenous appearance, yet a very laudable and well concocted pus appeared.

From this period to the 15th or 16th day after the accident, every thing seemed promising; the discharge was favourable, and granulations had taken place in the thigh, which began to heal; the same process had commenced in the wound of the ankle after the integuments had sloughed off; and, notwithstanding all these flattering appearances, strong spasms, with considerable rigidity of the muscles of the neck, face, chest, &c. took place; his jaw at length became locked, and the muscles of the thigh, leg and foot alternately were convulsed. Wine, brandy, and bark, the second a little diluted, were given freely; mercurial ointment was rubbed into his thighs and legs; calomel was also given, and opium freely administered; the dressings were now of digestive, and pledgets ex ol. terebinth, with a view to excite a greater inflammation and discharge, were applied, though this last was not much lessened when the spasms came on; notwithstanding all these means, and a ptyalism had come on, he died on the twenty-seventh day from the accident, and eleventh or twelfth from the attack of the locked jaw.* The wound of the

* From the fatal termination of this case, it may be suspected by some, that the solution applied here having a tendency to counteract the inflammation, accelerated the attack of the spasms; but how far this may be true it is not easy to determine. That tetanus more frequently occurs in lacerated wounds of the hands, feet, fingers, toes, &c. whether from fire arms, or other accidents; and this is perhaps the more likely where tendons, or rather nerves, are pretty much diffused, is very well known, and has always been remarked; and, for aught we know, has occurred under every form of external or even internal treatment. In the cases related by Mr. Carter, (Vide Medical Facts and Observations, vol. 6, p. 66,) nearly the whole of them were of the worst sort, and I think full as bad, if not worse, than this I have related. Nothing of this kind however took place, nor do I think we need be deterred from its use in similar cases. I should have observed, that from the third or fourth day, this patient was never free from symptomatic fever; and as every thing was used that might tend to keep up the vigour and energy of the system, as well as to excite the external parts to action from the very first appearance of the spasms, and his diet was never low or sparing, it will plainly appear, that I did not neglect to put in practice the plan of Dr. Rush, which has been said to have proved ingeneral more particularly successful than others.

the thigh was nearly healed; that of the ankle had begun to fill up near the astragalus and tarsus, but the part about the tendo Achilles appeared uncovered, and retained some matter in contact with it and the os calcis. As the articulation of the joint was sound, the tendo Achillis entire, and a free circulation between the leg and foot preserved, amputation could not have been justified from reasoning *a priori*; and to have performed it after the commencement of the spasms, would most probably have proved unsuccessful.

To many contused, lacerated, and even incised wounds, in different parts of the body, I have applied this solution, both as a lotion and dressing, on the head, face, limbs, &c. and that with superior success to any other application I had hitherto used. Prior, however, to the perusal of Mr. Carter's paper, I had never applied it where there was a wound, but confined it only to contusions, sprains, simple fractures, &c.; and although I have (except in some very particular cases) disused warm poultices, contenting myself with the diluted *aq. litharg. acetat.* as a lotion, and sometimes in form of a cold poultice, yet in this last form I have, except in some particular cases of inflamed tumefied breasts, proceeding from engorged milk, &c. scarcely used it at all.

Mr. Carter's solution, as a common dressing to lacerated wounds, is two drachms of crude sal ammoniac to half a pint of vinegar; this is an active and, I think, pretty strong application. I have, however, been under the necessity of sometimes diluting it with water; as, in cases where the wounds become tender after suppuration, and where they are bare from the separation of their sloughs, it is usual for me to add to it an eighth or tenth part of camphorated spirits more particularly as a dressing to the wounds, and that not only with a view of warming and invigorating the parts, but correcting the fætor usually attendant on their suppuration. The formula of the prescription runs thus: ℞. Sal ammon. crud. ʒij. aq. pura. ℥ij. acetum. com.* ℥ij. spt. vin. camph. ʒiij. m. ft. lotio pro usu. As in some cases, the lint wet with this composition, on drying, frequently

* Much, however, depends on the purity and strength of the vinegar used; for should it unfortunately happen to be mixed or adulterated with any of the mineral acids, to which it is liable when flat, its effects on wounds will not only be pernicious, but the pain and inflammation excited extremely dangerous.

quently sticks to the wounds and creates uneasiness, more especially when the discharge lessens, I have in these instances applied pledgets of lint, or tow, spread with cerate, above the dressings.

I shall now proceed to point out what relates to its use where there is no wound, and those cases where it may not be used, or where it may prove hurtful. In sprains, bruises, fractures, dislocations, and other recent injuries, its usefulness is already acknowledged. In these cases, however, it is preferable to the aq. saturn. spt. Mindereri, linim. sapon, and spt. vin. camph. For, although the first will diminish pain and swelling, it yet leaves a hardness; the second is less active, and, as well as the third and fourth, more expensive; and as the two last are not only more stimulating, whereby as primary applications they become improper, but from their active properties seem more likely to excite than diminish the inflammation already present, which the solution is calculated to suppress; hence they would appear more useful after diminution of the inflammatory symptoms; and when these are quite gone and the parts become debilitated, their tone and vigor may be more powerfully excited by the use of the volatile liniment with ol. terebinth, ol. terebinth c. camph. or bals. sulph. terebinth. The solution should be discontinued when the pain, inflammation and swelling are gone off, as its farther use appears no longer necessary.

By this regular mode of treatment, violent sprains, contusions and dislocations, have been very readily cured; which, in the very irregular and unskilful method of practice, by emollient poultices and other inconsistent applications, have continued not only long in hand, but frequently remained incurable.

It does not appear, as far as my experience extends, that this solution has proved useful in scrophulous affections, or white swellings of the knee or other joints, from the same cause, except some particular cases where there is great uneasiness from external violence, or pains in the parts proceeding from acute rheumatic or arthritic affections. In these last, although it has greatly lessened pain by diminishing the temperature of heat in the parts; yet the benefit derived from it here has not been permanent, and the sudden removal of the pain in one instance where a *metastasis* took place from its affecting the stomach, had very nearly proved fatal.

In sciatica or hip gout, and other cases of chronic rheumatism, matism, I have not known it produce any good effect, nor can it be expected to be useful or beneficial in œdematous swellings, paralysis, contractions of the tendons, muscles, or anchylosis.

In four cases of strangulated hernia, one of which was a female, I found this solution applied liberally (by wetting compresses of linen and applying it as cold as possible, constantly repeating the same till they became warm or dry) to succeed so effectually as lessened their bulk, whereby a reduction was effected. In two of these cases warm fomentations had been previously used, which, as is usually the case, aggravated the pain, and increased the size of the tumour.

In these last cases the solution was used in the proportion of half an ounce of sal ammon. crude to a pint of vinegar, mixed with an equal quantity of water.

These facts of its effects in such like cases are by no means unknown; as this or similar applications have been recommended by the late Mr. Pott and others. Ice has also been recommended, and used with success.

It has also by some surgeons been highly extolled in certain cases of incipient hydrocele; I think by Mr. Keate, who has, as I have been informed, asserted that it has performed radical cures. That this may be true is possible; but although I have given it trials in about four cases, two of which were only just forming, and pushed its use with vigour, applying it made pretty strong for above five or six weeks, and have also known it used in similar cases by others, yet I never knew it produce a radical cure. It has certainly diminished the volume or size of the tumour to less than half its bulk, but on leaving off, the application it always returned.

In the *cynanche parotidea*, or mumps, a certain epidemical distemper which sometimes affects one or more *testes* with inflammation, pain, and tumefaction, strongly resembling *hernia humoralis*, for which it has been mistaken, I have tried this solution with no small success as an application in several instances, of suddenly producing a repulsion, in the following reduced state, viz. ℞. Sal ammon. crud. ʒß. acet. ℥i. aq. pura ℥vij. spt. vin. camph. ʒi. ft. lotio. This was applied in a tepid state, and repeated frequently, by wetting in it compresses of thin linen rags.

When accidental bruises or sprains have occurred to produce tumefaction in either of the *testes*, I have used this solution in the form just prescribed with considerable advantage, advantage, although it has been deemed necessary in some instances to apply leeches. The *aq. saturnini* has also done good here; but on account of the hardness of the parts resulting from its use, this last remedy by me is justly preferred.

There is a certain species of head-ach which I have sometimes met with, not from a carious tooth or teeth, but where considerable external heat of the forehead, throbbing of the temporal arteries, and a painful sensation of the eyes and their coverings, have prevailed. This pain is often very extreme, and not unfrequently affects the system by increasing the frequency of the pulse. I do not mean a spasmodic or nervous affection of the head, or painful sensation of the face, &c. described by the late Dr. Fothergill. I say, in the first mentioned complaints, I have found this solution in the first prescribed form, sometimes diluted with one third part water, and at others, even in Mr. Carter's method, with the addition of some camphorated spirits, give great relief. I mean not that this solution is to be considered as capable of curing head-achs: these are often *idiopathic* diseases; but I consider the great relief that is frequently produced by applications of different kinds when this part is dreadfully pained, and which the patients call aloud for, as useful. To determine precisely the nature and cause of these affections is extremely difficult; and when we even succeed in procuring relief, or curing the disease, it is as often effected by random as by a *real* discernment; the feelings of the patient will always be the best guide; and where stimulating remedies are found to do no good, but harm, those of an opposite quality, such as the solution we have described, and varied in strength according to circumstances, will often prove of considerable benefit.

In some cases of delirium from fever, and in violent head-achs from drunkenness, where I have formerly prescribed vinegar, I have, however, found the solution much more powerful; and in ecchymosis, or lividnes of the eyelids from blows, &c. of all remedies I ever knew tried, this has the soonest taken off the blackness.

Many opportunities have occurred in which I have used with singular success this solution in *hernia humoralis*, but this was in the mild form, as recommended in the mumps. In full habits, strongly marked with symptomatic fever, general venesection *ad deliquiem animi* has always appeared to me the most decisive, prompt, effectual, and even safe remedy; but in those cases where the inflammation was local, I have

I have contented myself with applying leeches. These means, varied according to circumstances, with the occasional use of any mild laxatives, and opium where violent pain urges, given in clysters; which last method has frequently succeeded in preference to its exhibition in the usual form, not forgetting the horizontal posture, with the lotion already noticed, which I can affirm is superior to emollient poultices or *aq. saturn.* the general internal treatment being left to the discretion of the practitioner. It is necessary to remark, that when the pain goes off, and the swelling continues long and subsides slowly, *i. e.* when the inflammation is quite gone, stimulating it with the application of volatile liniment cum camph. very speedily reduces it to its natural size.

Phymosis and paraphymosis proceed not always from one and the same cause; the first, which in some persons is perfectly natural, is never considered of any consequence, unless the *preputium* or *glans* are in a diseased state, and which scarcely occurs but from venereal infection: the second, which is sometimes from the same cause, is however as frequently produced from the tightness of the *preputium,* when pressed over the *glans* by mere accident. The phymosis happening more frequently to come under our observation, and requiring such applications as tend to diminish inflammation and tumefaction, various have been the remedies proposed and used to effect this purpose. To enter into an inquiry respecting the different varieties and stages of this very troublesome and even dangerous complaint, would be only over-leaping the limits I had prescribed myself at my first setting out on this paper, and which I am afraid is already too much extended. Suffice it then to say, that this solution in the mild form, and which may be varied according to circumstances, is infinitely more efficacious as an external application on the exterior surface of the penis, than the *aq. saturnini,* both of which I have often used. I cannot, however, recommend either of them as injections between the *glans* and *preputium,* which are usually more or less in a state of excoriation or ulceration; but where the exterior covering of the penis is free from any solution of continuity, and the diseased state of the *glans* and *preputium* is not of long standing, and the latter not being indurated, this very lotion has, by applying it cold with cloths dipped therein, and frequently repeated, often proved extremely beneficial in diminishing the inflammation, so as frequently to effect the uncovering of the

 *glans,*

*glans*, whereby the mischief is more in our power. But where the *preputium* and *glans* are in a hardened callous situation, from the long continuance of the ulcerated state of both these surfaces, a discharge should be encouraged by the application of emollient cataplasms, warm fomentations, and mild injections of barley water and milk, to keep the parts clean and encourage it; the increase of which, in this instance, rarely fails to lessen the tumefaction. The external treatment on which the cure greatly depends, is left to the discretion of the prescriber. Of paraphymosis I can only say, that from whatever cause it may originate, it must if possible be reduced by manual operation; in any other way it cannot: but to describe this and the chirurgical, would be unnecessary; yet when its reduction is once effected, I know of nothing that will diminish the enlargement of the prepuce so readily as the solution.

Thus far I have extended these observations, which, as I before remarked, are certainly more minute and diffusive than I at first intended, and what may appear unnecessary to veterans in medical science; yet, as they will be read by others who may be inexperienced, and at a loss how to discriminate its use with accuracy, the apology I have here offered will, I trust, sufficiently plead my exculpation.

*Sunderland, Oct. 27, 1801.*

